# Plasma membrane-derived microvesicles released from tip endothelial cells during vascular sprouting

**DOI:** 10.1007/s10456-012-9292-y

**Published:** 2012-08-11

**Authors:** Daniela Virgintino, Marco Rizzi, Mariella Errede, Maurizio Strippoli, Francesco Girolamo, Mirella Bertossi, Luisa Roncali

**Affiliations:** Human Anatomy and Histology Unit, Department of Basic Medical Sciences, University of Bari School of Medicine, Piazza Giulio Cesare, Policlinico, 70124 Bari, Italy

**Keywords:** Neuroangiogenesis, Tip cells, Endothelial filopodia, Plasma membrane-derived microvesicles, Human foetal brain, Immunofluorescence confocal microscopy

## Abstract

During human foetal brain vascularization, activated CD31^+^/CD105^+^ endothelial cells are characterized by the emission of filopodial processes which also decorate the advancing tip of the vascular sprout. Together with filopodia, both the markers also reveal a number of plasma membrane-derived microvesicles (MVs) which are concentrated around the tip cell tuft of processes. At this site, MVs appear in tight contact with endothelial filopodia and follow these long processes, advancing into the surrounding neuropil to a possible cell target. These observations suggest that, like shedding vesicles of many other cell types that deliver signalling molecules and play a role in cell-to-cell communication, MVs sent out from endothelial tip cells could be involved in tip cell guidance and/or act on target cells, regulating cell-to-cell mutual recognition during vessel sprouting and final anastomosis. The results also suggest a new role for tip cell filopodia as conveyor processes for transporting MVs far from the cell of origin in a controlled microenvironment. Additional studies focused on the identification of MV content are needed to ultimately clarify the significance of tip cell MVs during human brain vascularization.

**Electronic supplementary material:**

The online version of this article (doi:10.1007/s10456-012-9292-y) contains supplementary material, which is available to authorized users.

## Introduction

Exosomes and other plasma membrane-derived vesicles, known as ectosomes, shed vesicles, or microvesicles (MV), are secreted by many different cell types and are thought to play important roles in cell–cell communication by releasing various active molecules including signalling molecules, mRNAs, and miRNAs, in both physiological and pathological processes. In blood, as ‘microparticles’, in seminal fluid, as ‘prostasomes’, or within the tissue microenviroment, plasma membrane-derived MVs bridge the space between distant cells, having a role in cell-to-cell communication during development as well as in inflammatory responses and tumour growth. At the the recipient cell surface, shed MVs find specific ligands, become internalized and enter the endocytic pathway or directly fuse with the plasma membrane and release their content within the cytoplasm. Unlike exosomes that form in endosomal compartments and have a diameter size of 100 nm or less, microvesicles have a larger diameter, from 100 to 1.000 nm, and directly originate from plasma membrane protrusions, then detach from the cell surface (reviewed in [1–3]).

Recent studies ‘in vitro’ and ‘in vivo’ have demonstrated that platelet- and tumour cell-derived MVs can participate in angiogenic events by transferring a series of proangiogenic factors, including growth factors (VEGF, bFGF, PDGF), chemokine receptors (CCR5, CXCR4), and matrix metalloproteinases (MMP2, MMP9), which all have endothelial cells and/or endothelial progenitor cells (EPCs) as target cells and contribute to regulate vessel repair, sprouting, and invasiveness by inducing intracellular signalling, following binding to receptors or by genetic transfer (reviewed in [4, 5]). After their internalization in endothelial cells, MVs derived from EPCs promote angiogenesis, activating the formation of capillary-like structures through the PI3 K/AKT signalling pathway [6]. Tumour cell shedding of MVs is positively correlated with malignancy and induces endothelial cell migration and ‘in vivo’ angiogenesis via vesicle-associated sphingomyelin [7]. Also exosomes produced ‘in vitro’ by K562 chronic myeloid leukemia cells trigger angiotube formation in human umbilical endothelial cells (HUVECs) [8, 9] and hypoxic glioblastoma cells elicit an angiogenic endothelial cell phenotype by releasing an MV-associated tissue factor involved in activation of the PAR-2/heparin-binding EGF-like growth factor [10]. Endothelial cells themselves can also release MVs, thus contributing together with other stimuli to the angiogenic activity of growing microvessels. Under VEGF and bFGF stimuli, cultured HUVECs shed MMP/TIMP-containing MVs and are possibly involved in the autocrine control of vessel growth [11]. This study, carried out in developing human foetal brain by using high resolution confocal microscopy and the endothelial markers CD31 and CD105, reveals shedding of CD31^+^/CD105^+^ MVs by endothelial cells of growing microvessels and the presence of filopodia and associated MVs at the advancing front of the sprout, where they may form a conveyor/messenger apparatus of endothelial tip cells, possibly involved in the process of vascular growth, branching and anastomosis.

## Materials and methods

### Histology

Autopsy specimens of telencephalon were collected from 2 legally aborted foetuses (9–10 weeks of gestation, wg) and 2 foetuses spontaneously aborted due to preterm rupture of the placental membranes (22 wg), with no history of neurological pathologies. Permission to collect tissue was obtained from the maternal donor at the end of the abortion procedure. The sampling and handling of human specimens conformed to the ethical rules of the Department of Pathology, Medical School, University of Bari, Italy, and approval was gained from the local Ethics Committee of the National Health System in compliance with the principles stated in the Declaration of Helsinki. The foetal age was estimated on the basis of the crown-rump length and/or pregnancy records (counting from the last menstrual period). At autopsy, the selected foetuses did not reveal macroscopic structural abnormalities and/or malformations of the central nervous system. The dorso-lateral wall of the telencephalic vesicles (future neocortex) was dissected and fixed for 2–3 h at 4 °C by immersion in 2 % paraformaldehyde plus 0.2 % glutaraldehyde solution. Specimens were then washed in phosphate buffered saline (PBS, pH 7.6) and serially sectioned by a vibrating microtome. 20-μm sections were collected at regular intervals and processed for conventional histological analysis with toluidine blue staining to ascertain the absence of microscopic malformations; all the other sections were stored in PBS plus 0.02 % paraformaldehyde for fluorescence immunolabeling.

### Immunofluorescence and laser confocal microscopy

Single and multiple immunostainings were carried out with the following primary antibodies: mouse IgG_1k_ anti-Human CD31 (diluted 1:10 in blocking buffer-PBS, 1 % bovine serum albumin, 2 % FCS-BB; Dako Cytomation, Glostrup, Denmark), rabbit polyclonal IgG anti-Human CD105 (prediluted; Abcam, Cambridge, UK), rabbit polyclonal IgG anti-NG2 (diluted 1:50 in BB; this affinity purified antibody directed against the D2 domain of NG2 was a gift from W. Stallcup, The Burnham Institute for Medical Research, La Jolla, CA, USA), and mouse monoclonal IgG_1k_ anti-Human collagen type IV (diluted 1:100 in BB; Dako). After allowing the sections to adhere on polylysine slides (Menzel-Glaser, GmbH, Braunschweig, Germany) by drying for 10 min at room temperature (RT), they were submitted to the following protocols: rehydration with PBS for 5 min at RT or microwave pre-treatment in 0.01 M citrate buffer (pH 6.0) for 15 min at 750 W (for CD105); incubation with 0.5 % Triton X-100 in PBS for 30 min at RT and with BB 30 min at RT; incubation with diluted single or combined primary antibodies overnight at 4 °C; incubation with appropriate fluorophore-conjugated secondary antibodies (anti-rabbit 555 and anti-mouse 555 diluted 1:400 in BB; Invitrogen, Eugene, OR, USA) or biotinylated secondary antibodies (diluted 1:400 in BB; Vector Laboratories Inc.; Burlingame, CA, USA) followed by fluorophore-conjugated streptavidin (488; diluted 1:400 in BB for 45 min at RT; Invitrogen). After immunolabelling, the sections were fixed in 4 % PFA for 10 min and counterstained with TO-PRO3 diluted 1:10 K in PBS for 5 min at RT (633; Invitrogen). After each incubation step the sections were washed 3 times for 5 min with PBS. Finally, the sections were coverslipped with Vectashield (Vector) and sealed with nail varnish. Negative controls were prepared by omitting the primary antibodies, pre-adsorbing the primary antibodies with an excess of antigen when available, and mismatching the secondary antibodies. The stainings were examined under the Leica TCS SP5 confocal laser-scanning microscope (Leica Microsystems, Mannheim, Germany) using a sequential scan procedure. Confocal images were taken at 250 nm intervals through the z-axis of the sections. Confocal images were taken with 40× and 63× oil lenses. Z-stacks of serial optical planes (projection images) and single optical planes were analyzed by Leica confocal software (Multicolour Package; Leica Microsystems). The size of plasma-membrane vesicles was evaluated with LAS-AF SP5 software (Leica Microsystems) on 63× magnification fields zoomed 3 times. Microvesicles diameter (nm) was measured on single optical planes from CD31- and CD105-labeled sections (n = 8, 9–10 wg; n = 15, 22 wg) for a total of 55 tip cell fields. The results were expressed as mean ± SD.

### Transmission electron microscopy

Small samples collected from the previously described telencephalon specimens were submitted to electron microscopy procedures. Briefly, samples were fixed in 0.1 M phosphate-buffered 3 % glutaraldehyde, post-fixed in phosphate-buffered 1 % OsO_4_, dehydrated in serial alcohols, and embedded in Epon 812. Ultrathin sections were cut with an LKB V ultramicrotome, stained with uranyl acetate and lead citrate, and observed under a CM 10 Philips electron microscope.

## Results and discussion

**Fig. 1:**
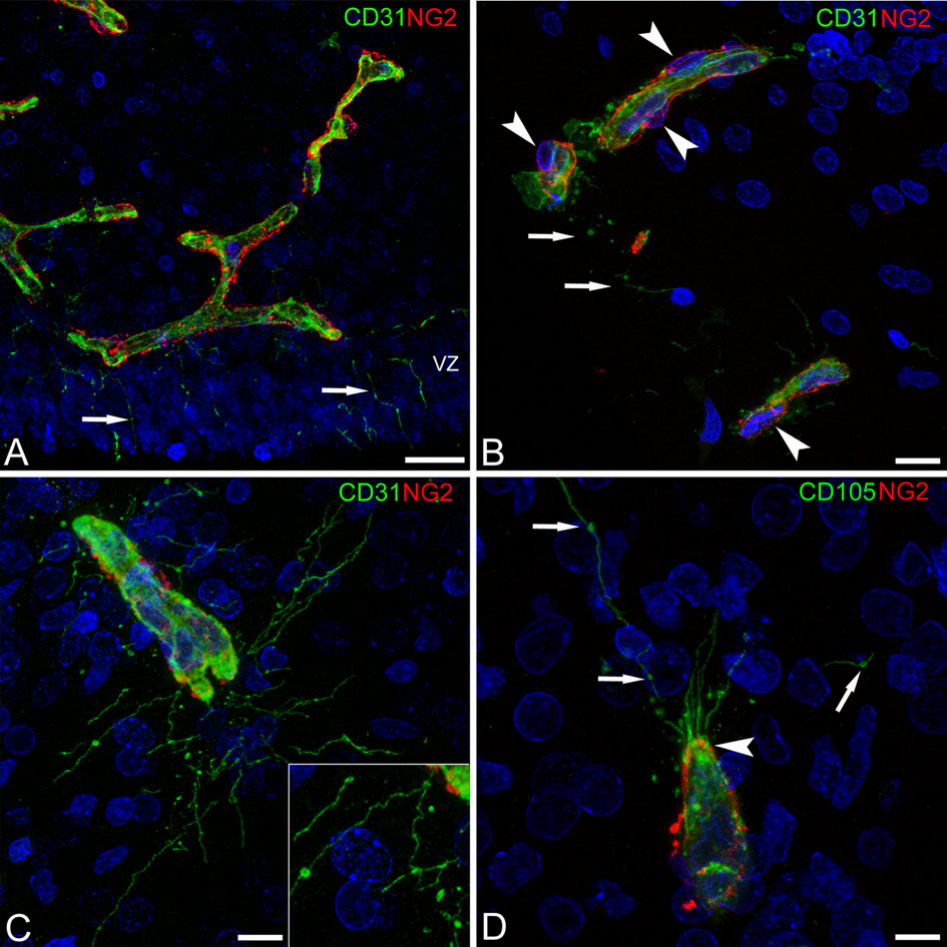
Confocal microscopy image of human foetal telencephalon at 9–10 wg, double labelled with CD31/NG2 (**A**–**C**) and CD105/NG2 (**D**). **A** Periventricular microvessels close to the ventricular zone (VZ) show CD31^+^ endothelial cells surrounded by activated, NG2^+^ pericytes; endothelial filopodia are sent out to the ventricle (*arrows*). **B** Two microvessels with well distinguishable NG2^+^ pericytes (*arrowheads*) and filopodia and MVs recognizable in the neuropil between them (*arrows*). **C** A growing sprout is shown with well developed spider web-like filopodia and a high number of associated MVs (see also the *inset*). **D** A typical CD105^+^ endothelial tip cell with a tuft of filopodia that pass through the NG2 cover (*arrowhead*) and appear associated to MVs at various distances from the sprout tip front (*arrows*). *Scale bars* = **A**, **C** 10 μm; **B** 15 μm; **D** 7.5 μm

**Fig. 2:**
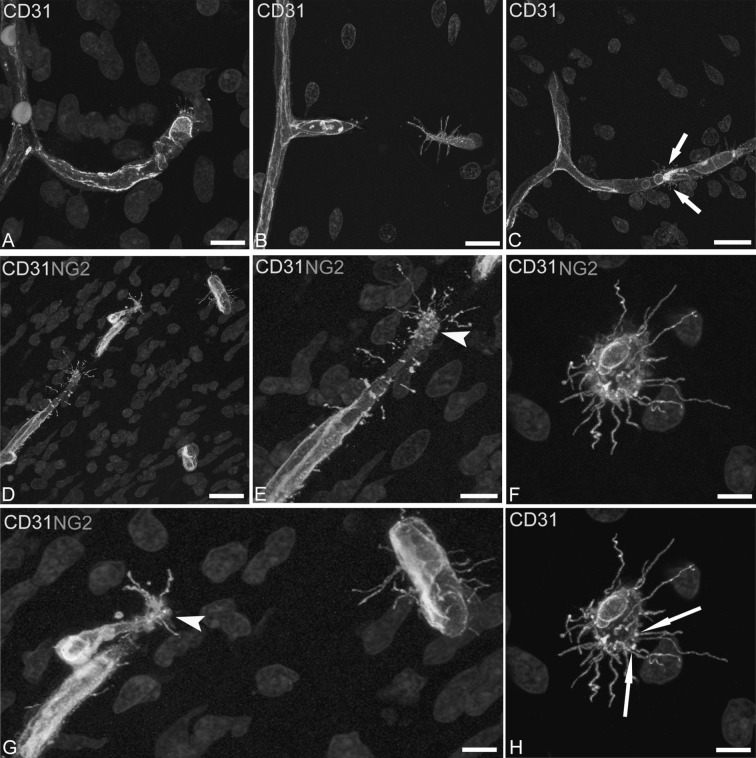
Confocal microscopy image of human foetal telencephalon at 22 wg, single labelled with CD31 (**A**–**C**, **H**) and double labelled with CD31/NG2 (**D**–**G**). **A**–**C** Different steps in vascular sprout growth and elongation (**A**) and in head-to-head vessel sprouting (**B**, **C**); note in **C** the fusion of two sprouts (*arrows*). **D, E, G** CD31 labelled growing microvessels enveloped by NG2^+^ pericytes; in **E** and **G** (two enlargements of picture **D**) sprout tips decorated by numerous filopodial processes (*arrowheads*). **F**, **H** Frontal view of the sprouting tip cell with filopodia and MVs, the latter also associated (**H**, *green channel* of **F**) with the filopodia base (*arrows*). *Scale bars* **A**, **E** 10 μm; **B** 15 μm; **C**, **D** 20 μm; **F**, **H** 7.5 μm

At 9–10 weeks of gestation (wg), when the cerebral cortex is formed by only few rows of neuroblasts (the pre-plate), branches of the earliest penetrated telencephalic microvessels lie at the border between the ventricular zone (VZ) and the subventricular zone (SVZ), forming a primary periventricular plexus. These microvessels are distinctly labelled by the endothelial marker CD31 and also show a discontinuous pericyte layer revealed by the pericyte-specific marker NG2 (Fig. 1). Endothelial long filopodial processes arise from the ventricular side of these microvessels, run right through the cells of the VZ and abut the ventricular telencephalic surface (Fig. 1A). Throughout the telencephalon wall, growing microvessels show active endothelia characterized by long filopodia and abluminal plasma membrane protrusions; membrane-derived MVs are also recognizable and appear associated with the filopodial processes (Fig. 1B, C). At this time, telencephalon microvessels emanate a few vessel sprouts which are revealed by typical CD31^+^/CD105^+^ endothelial tip cells, with abundant, long filopodial extensions and CD31/CD105 labelled MVs (Fig. 1C, D). Later on, at 22 wg, as a consequence of brain size increase and cerebral cortex development, an active phase of vascular branching is seen to start, firstly in the subcortical brain layers. At this time, the CD31 staining pattern is junctional in parental vessels and at the origin of the sprout stalk, whereas it is diffusely cytoplasmic at the sprout ending and marks tip cells together with tufts of filopodial processes and associated MVs (Fig. 2A–C). On favourably oriented sections, head-to-head vessel sprouts are recognizable at various steps of the process growth journey, while they grow to fill the gap between their respective parental vessels, and finally fuse head-to-head to form a new, patent, anastomotic collateral (Fig. 2A–C) (see also Online Resource 1).

**Fig. 3:**
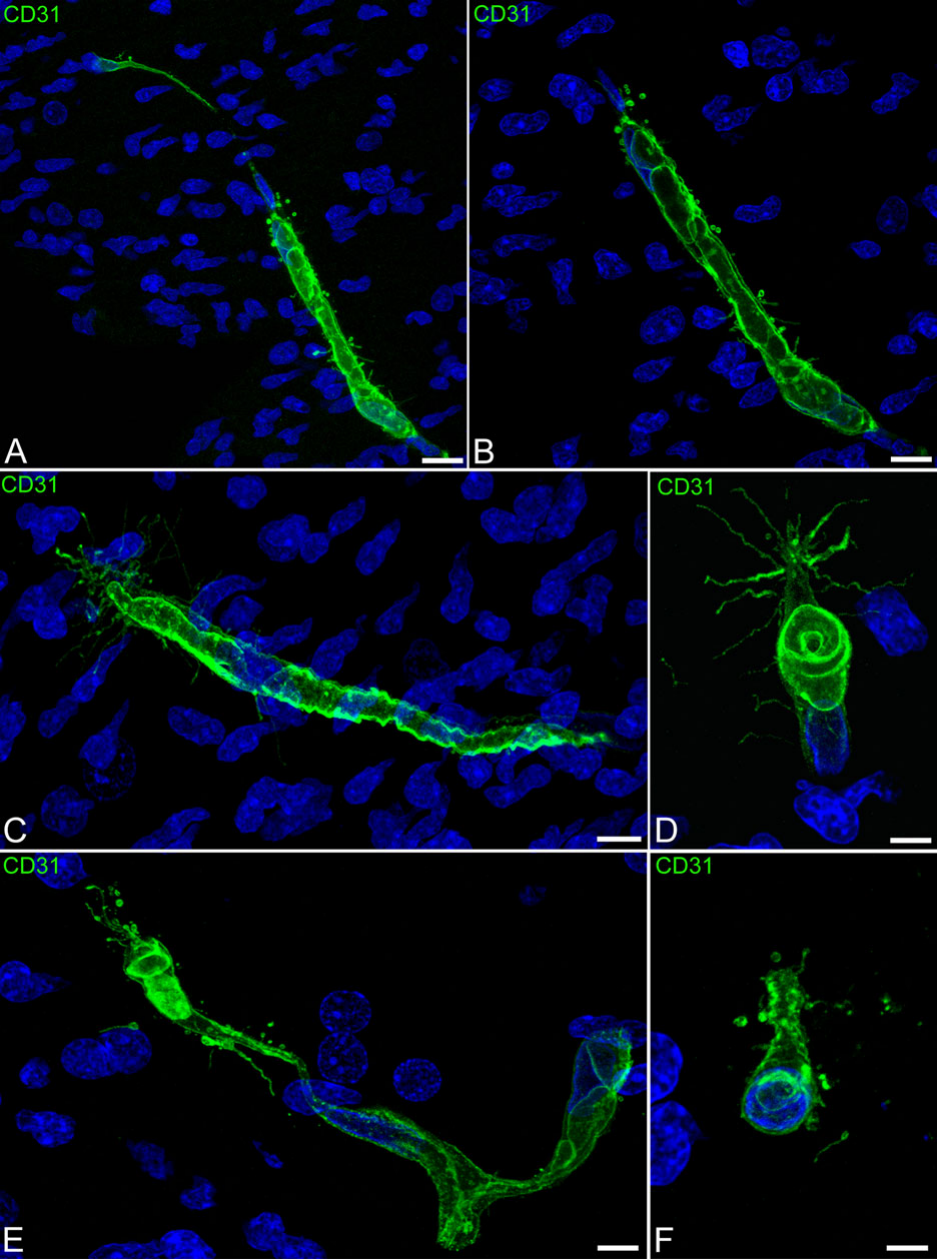
Confocal microscopy image of human foetal telencephalon at 22 wg, single labeled with CD31. Short filopodia and isolated MVs are released from the entire length of the vessel stalk, whereas long filopodia and numerous MVs originate from the tip cell; relationships of MVs and vessel wall are better shown by a zoomed single optical plane (**B**) from the stack of optical planes (**A**). *Scale bars* **A** 15 μm; **B**, **C** 10 μm; **D**, **F** 5 μm; **E** 7.5 μm

**Fig. 4:**
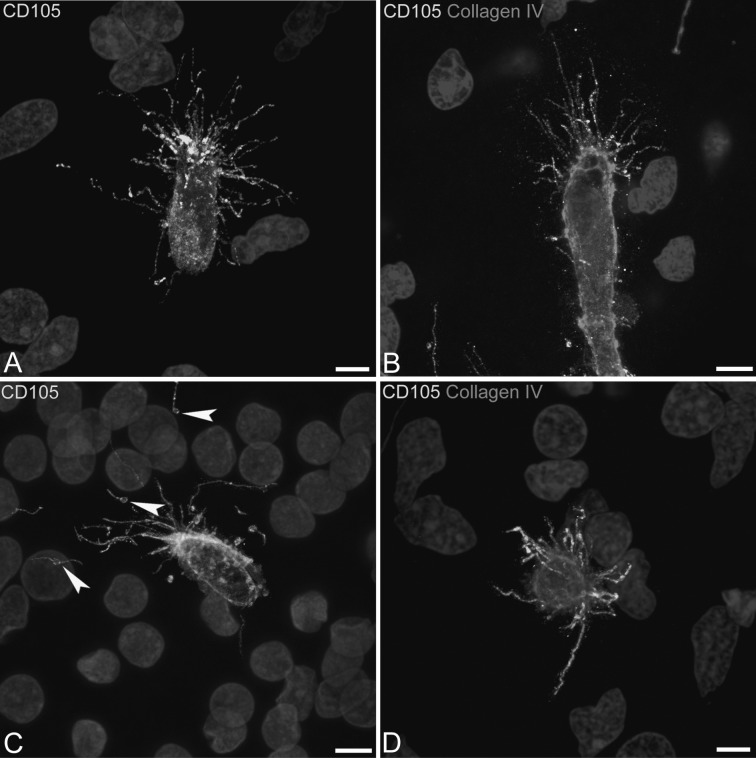
Confocal microscopy image of human foetal telencephalon at 22 wg, single labelled with CD105 (**A**, **C**) and double labelled with CD105/collagen IV (**B**, **D**). **A**, **C** Tip cells, tufts of filopodia and associated MVs are revealed by the endothelial proliferation marker CD105; note in **C** shed MVs associated with filopodia far from the tip cell (*arrowheads*). **B**, **D** The profile (**B**) and the frontal view (**D**) of CD105^+^ endothelial filopodia and MVs, which emerge from the collagen IV basal lamina. *Scale bars* **A**–**D** 5 μm

On double labelled CD31/NG2 sections, growing microvessels appear enveloped by NG2^+^ pericytes (Fig. 2D–G) and the target-oriented filopodia emerge from the pericyte envelope, and elongate in the surrounding neuropil with accompanying MVs (Fig. 2D–G). At the sprout tip MVs bud off from the endothelial tip cell plasma membrane close to the filopodial base, and maintain this contact at a distance from their origin throughout the filopodial length (Fig. 2F, H). An accurate analysis of endothelial-derived filopodia/MVs shows further details on these cellular apparatus, a few MVs and short filopodia being recognizable along the sprout stalk formed by activated, proliferating endothelial cells, whereas a larger number of MVs associated with numerous, long filopodial extensions are concentrated around the tip endothelial cells (Fig. 3A–F). The growing sprouts are also revealed by endoglin/CD105, that reveals angiogenically activated endothelial tip cells, their filopodia and associated MVs (Fig. 4A, C). On double immunolabellings with CD105 and collagen IV, the tip cell endothelial filopodia/MVs apparatus is free from the remaining collagen IV basement membrane and projects into the surrounding neuropil (Fig. 4B, D). Tip cell MVs labelled by both CD31 and CD105 have an estimated mean diameter of 717.02 ± 130.01 nm (743.64 ± 138.93 at 9–10 wg and 702.12 ± 125.16 at 22 wg) measured on single optical planes from projection stacks at suitable intervals, as shown in the animation (Online Resource 2).

**Fig. 5:**
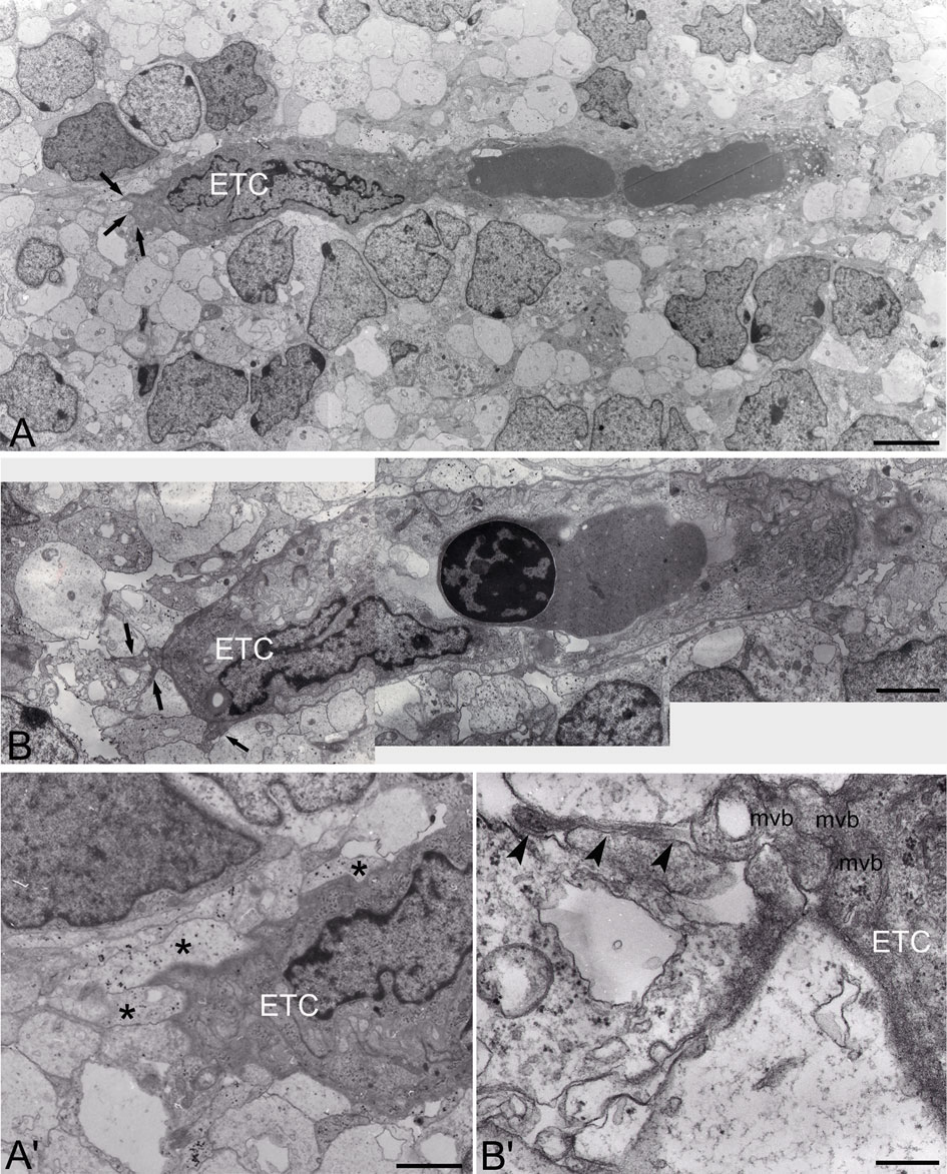
Transmission electron microscopy of human foetal telencephalon at 9–10 wg. **A**, **B** Two vascular sprouts with endothelial tip cells (ETC.) and emerging filopodial processes (*arrows*); note in **B** a nucleated blood cell within the narrow vascular lumen. **A’**, **B’** Higher magnification of vascular sprouts (depicted in **A** and **B**) shows details of endothelial tip cells (ETC.): **A’** filopodial emergence and tip cell-contacting astroglia endfeet (*asterisks*), **B’** multivesicular body-like structures (mvb) at the base of a drumstick shaped filopodial process (*arrowheads*). *Scale bars* **A** 5 μm; **B** 2 μm; **A**’ 1.5 μm; **B**’ 400 nm

Ultrastructural appearance of endothelial tip cells and filopodia is revealed by TEM analysis (Fig. 5). The luminal and the abluminal sides of vascular sprouts are irregular and the vessel lumen is narrow or absent; the endothelial cells are characterized by electron-dense cytoplasm, the tip cell large nucleus is positioned at the leading front (Fig. 5A, B). At this site, filopodial processes rich in bundles of microfilaments are seen to emerge from a large base, which contains multivesicular body-like structures and polysomes (Fig. 5A′, B′). Perivascular astroglia endfeet, revealed by their electron-lucent cytoplasm containing glycogen granules, are seen in contact with endothelial tip cells (Fig. 5A′, B′).

Angiogenesis is well known to be under the control of several growth factors, receptors, co-factors and enzyme systems, all involved in a complex and finely tuned balance between pro- and anti-angiogenic effects (reviewed in [12–16]). At the beginning of cerebral cortex vascularization, early penetrating microvessels elongate and start to put out primary branches close to the VZ, subsequent waves of radial microvessels penetrate the nerve wall and at midgestation a remarkable phase of vessel branching is revealed by the presence of several sites of vascular budding and sprouting in the subcortical layers. The results show that during these phases of foetal brain vascularization activated endothelial cells are characterized by filopodial processes and plasma membrane-derived MVs. At the sprouting front, endothelial tip cells send out CD31/CD105 MVs, which appear to run free in the tip cell vicinity or to be associated with endothelial filopodia. This conveyor/messenger tip cell apparatus may be involved in the process of vascular growth, branching and anastomosis, transporting quanta of active molecules along a controlled path towards a vascular target. According to this hypothesis, the observed end-to-end mode of branching could be mutually regulated, vessel sprouts being able to orientate one another by shedding MVs associated to guiding filopodia. A possible mechanism of ‘tentacle recognition’ was firstly described by Thomas Bär, on the basis of his observations on ink-injected specimens and electron microscopy preparations, and illustrated in a diagram (kindly provided by Prof. J.R. Wolff), where he described “A fusion of capillary sprouts with preexisting capillaries or with another sprout may be initiated by contact of small endothelial tentacles” [17] and Online Resource 3. Recently, tip cells that meet and fuse, forming capillary loops, have been revealed by time-lapse confocal microscopy [18]. In this scenario, shed vesicles may mediate multiple biological processes by horizontal transfer of proteins and RNAs [19], by releasing their molecular content close to or directly into the recipient cells, endothelial and/or perivascular cells (pericytes, astrocytes), or by delivering membrane-associated molecules, as in the case of endoglin/CD105 that, through interactions with the TGF-β receptors, affects TGF-β responses, finally modulating endothelial cell proliferation, differentiation, and migration (reviewed in [20]). The detection at the tip cell leading edge of multivesicular bodies, which have been suggested to be involved in the origin of cell membrane-derived vesicular structures (reviewed in [21]), supports the idea that an heterogeneous population of membrane vesicles, included the described MVs associated with tip cell filopodia, may represent a component of a specialized tip cell site for cell-to-cell communication. After these preliminary observations carried out on human brain vascularization, we can only presume that similar mechanisms may be involved in angiogenesis in different CNS regions and in other species since, to the best of our knowledge, MVs associated to endothelial tip cell filopodia have been barely described. In developing retina, one of the most widely used models for investigation of CNS physiological and pathological angiogenesis, characteristic swellings similar to membrane-associated MVs have been shown at the end of tip cell filopodial extension in VEGF120 transgenic mice [22].

The next goal of this research project will be to devise immunohistochemical/confocal and electron microscopy protocols for ‘in situ’ testing of a number of growth factors and related molecules involved in MV-mediated cell-to-cell communication during normal human brain angiogenesis.

## Electronic supplementary material

Below is the link to the electronic supplementary material.

Supplementary material 1 (AVI 95236 kb). Online Resource 1. A newly-formed anastomosis originating from fusion of two vessel sprouts. Double immunolabelling of endothelial cells with a rabbit pAb anti-Glut-1 (1:50 dilution; Chemicon; Temecula, CA, USA) and astrocytes with a mouse mAb anti-GFAP (1:100 dilution, DakoCytomation, Glostrup, Denmark)

Supplementary material 2 (AVI 39940 kb). Online Resource. 2. Shedding of CD31 + endothelial membrane-derived MVs visualized on the sequence of single optical planes

Supplementary material 3 (TIFF 12725 kb). Online Resource 3. A typical, initial vascular sprout shown by horseradish peroxidase injection during chick embryo optic tectum development (Roncali et al., Acta Neuropathol. 70:193–201, 1986 and unpublished observations)
