# Supplementary figures and images for: Plasma membrane-derived microvesicles released from tip endothelial cells during vascular sprouting
Source: Angiogenesis. 2012 Aug 11;15(4):761–9. doi: 10.1007/s10456-012-9292-y (PMC3496552; doi:10.1007/s10456-012-9292-y)

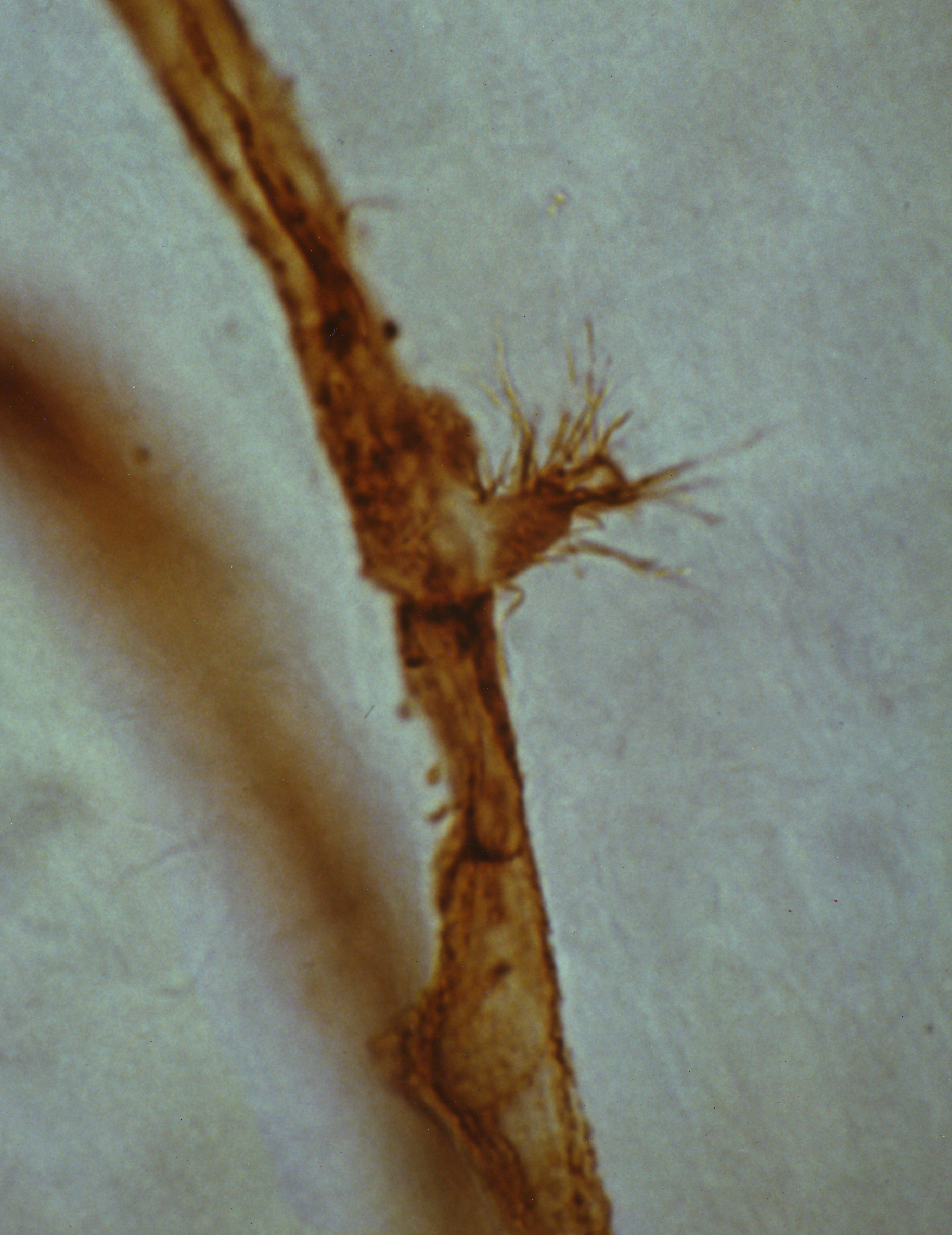

Supplement: Supplementary file 3 — Supplementary material 3 (TIFF 12725 kb). Online Resource 3. A typical, initial vascular sprout shown by horseradish peroxidase injection during chick embryo optic tectum development (Roncali et al., Acta Neuropathol. 70:193–201, 1986 and unpublished observations) [file 10456_2012_9292_MOESM3_ESM.tif]
